# Adaptive Stress Coping in Awake Bruxism

**DOI:** 10.3389/fneur.2020.564431

**Published:** 2020-12-09

**Authors:** Xabier Ander Soto-Goñi, Francisco Alen, Leticia Buiza-González, Danielle Marcolino-Cruz, Teresa Sánchez-Sánchez, Ignacio Ardizone-García, Fernando Aneiros-López, Laura Jiménez-Ortega

**Affiliations:** ^1^Department of Psychobiology and Behavioral Sciences Methods, Faculty of Odontology, Complutense University of Madrid, Madrid, Spain; ^2^Department of Conservative and Prosthetic Dentistry, Faculty of Odontology, Complutense University of Madrid, Madrid, Spain; ^3^Centre for Human Evolution and Behaviour, Instituto de Salud Carlos III, Complutense University of Madrid (UCM-ISCIII), Madrid, Spain

**Keywords:** bruxism, anxiety, temporomandibular disorder (TMD), psichological factors, neuroticism, stress coping

## Abstract

Numerous studies have analyzed the relationship between psychological factors and bruxism. However, the data are often obscured by the lack of precise diagnostic criteria and the variety of the psychological questionnaires used. The purpose of this study is to determine the association between awake bruxism and psychological factors (anxiety, depression, sociability, stress coping, and personality traits). With this aim, 68 participants (13 males) completed a battery of psychological questionnaires, a self-reported bruxism questionnaire, and a clinical examination. Based on their scores on the bruxism questionnaire and the clinical examination, subjects were divided into two groups. Subjects who met the criteria for “probable awake bruxism” were assigned to the case group (*n* = 29, five males). The control group (*n* = 39, nine males) was composed of subjects who showed no signs or symptoms of bruxism in the examination nor in the questionnaire. The probable awake bruxism group presented significantly higher levels of trait and state anxiety, symptoms of somatization, and neuroticism than the control group. Despite this, and when their problem coping strategies were considered, awake bruxers showed higher levels in Positive Reappraisal (*p* < 0.05), a strategy generally considered as adaptive. In conclusion, although awake bruxers in our study showed larger levels of anxiety, somatization, and neuroticism, they also displayed more adapted coping strategies, while according to previous data TMD patients (which generally also present high levels of anxiety, somatization and neuroticism) might tend to present less adaptive coping styles. Thus, awake bruxism may play a positive role in stress coping, which would be compatible with the hypothesis of mastication as a means of relieving psychological tension. This finding should be further confirmed by future research comparing TMD patients with definitive awake bruxers and controls and using larger and more representative samples.

## Introduction

Awake bruxism is a masticatory muscle activity during wakefulness that is characterized by repetitive or sustained tooth contact and/or by bracing or thrusting of the mandible. Sleep bruxism is a masticatory muscle activity during sleep that is characterized as either rhythmic (phasic) or non-rhythmic (tonic) (1). Mixed episodes are formed by awake and sleep bruxism. Recent studies found a prevalence of 5.0% for awake bruxism and of 16.5% for sleep bruxism (2). However, its prevalence among the young college population is much higher, reaching 37.9% for awake bruxism and 31.8% for sleep bruxism (3). Recently, it has been argued that the mere presence of awake or sleep bruxism should not be considered pathological on its own in otherwise healthy individuals, but rather as a risk for other negative health consequences. Moreover, in some individuals, it could even have positive consequences for the bruxer (e.g., mediating the recovery from respiratory arousals or reducing teeth wear due to gastro-esophageal reflux by increasing salivation) (1).

Patients with awake bruxism are more likely to experience jaw pain and/or limitations of movement than patients with sleep bruxism, which highlights the importance of considering this distinction (4, 5). Furthermore, the etiology of bruxism is not completely clear. While it is thought that morphological and pathophysiological factors may be related to bruxism, the importance of psychosocial factors in its etiology is becoming clearer, particularly in the case of awake bruxism (6, 7). Indeed, psychological and social factors appear to be critical in the transition from non-symptomatic bruxism or teeth clenching to a painful disorder (8). Thus, traditional approaches centered on occlusal interventions are being replaced by more comprehensive approaches which place special emphasis on psychosocial factors which are considered by some authors to be the most important etiopathological factor in bruxism (8–10).

Among the psychological factors influencing bruxism, a recent exhaustive review singles out anxiety, sensitivity to stress, depression, and some personality characteristics while pointing out that the impact of these factors has been clearly demonstrated for awake bruxism, whereas evidence linking psychosocial factors and sleep bruxism is less clear (7). Thus, an accurate diagnosis of bruxism, along with a clear distinction between awake and sleep bruxism, appears of paramount importance. In addition, more detailed investigations underline the importance of perceived stress in relation to bruxism (1, 11). Furthermore, perceived stress is strongly related to poor stress coping strategies (12). Generally, stress-related coping can be defined as the predictable cognitive and behavioral efforts to manage environmental and internal demands or conflicts (13), and data point out that stress coping influences perceived stress, anxiety and depression, among other factors (14, 15). Maladaptive coping strategies have, in turn, been linked to negative pain experiences in TMD (10). However, there is a lack of studies exploring the role of different stress coping strategies in bruxism.

Therefore, the objective of this study was to investigate the role of coping in awake bruxism by studying the following psychological factors: depression, anxiety, stress, coping styles, and personality traits.

## Materials and Methods

### Subjects

A total of 68 students (14 males, 54 females) were selected from a pool of 109 students who volunteered to participate in this study at the faculty of Dentistry, Complutense University of Madrid. The age of participants ranged between 17 and 31 (mean age = 19.6 years, SD = 2.6). All subjects underwent a clinical exploration and completed a self-assessed bruxism questionnaire. The case group (*n* = 29, 5 males, mean age = 20.0, SD =3.4), was composed of participants classified as awake bruxers in the clinical examination and the bruxism questionnaire (see materials section), but did not fulfill TMD criteria. Taken together, the case group met criteria for diagnosis of probable awake bruxism, according to the recent international consensus (16). The control group (*n* = 39, nine males, mean age = 19.2, SD =1.8) was composed of participants who did not present symptoms either signs of awake bruxism nor sleep bruxism in the clinical examination, and who showed no self-reported bruxism in the questionnaire. Student *t* analyses did not detect significant differences in the mean age between awake bruxism and control groups [*t*_(66)_ = 1.23, *p* = 0.22]. The number of males and females in the two groups could not be exactly matched (23 and 17.2% of males for control and case groups, respectively). However, a gender proportion analyses did not produce significant differences when comparing the ratio of males to females per group between the case and control groups (χ^2^ = 0.34, *p* = 0.76). Participants suffering from TMD and/or showing inconsistent results in the clinical exploration and self-assessment questionnaire were excluded. Among participants in the awake bruxism group, 16 (three males) presented probable sleep bruxism according to bruxism questionnaire and 15 participants (three males) presented mild tenderness to palpation (1 kg) but did not meet DC/TMD local myalgia criteria.

All participants were duly informed and gave their consent, the study has the approval of the ethics committee of the “Hospital Clínico Universitario,” UCM, Madrid, Spain (Reference: 12/043-E).

### Materials

#### Psychological Questionnaires

The battery of selected questionnaires included: the State and Trait Anxiety Inventory (STAI), the State and Trait Depression Inventory (ST-DEP), the Brief Symptom Inventory: Anxiety, Depression and Somatization (BSI-18), the Coping Response Inventory—Adult form (CRI-A), and the NEO Personality Inventory (NEO-FFI). All used questionnaires have high levels of reliability and validity in all their scales (>0.8) (17–21) and have been largely used in research [e.g., (22–24)].

Anxiety was measured using the STAI (17). It is composed of 10 items assessing state anxiety STAI-E (transient emotional state) and another 10 items for trait anxiety STAI-R (anxious, relatively stable propensity of the participant in general). The ST-DEP was used to assess depression (18). This 20-items questionnaire has a construction similar to the STAI, includes depression scales for state and trait depression, and within each one includes two euthymia and dysthymia subscales.

In addition, to further assess symptoms of depression, anxiety and somatization we included the BSI-18 questionnaire (19), which is a short questionnaire consisting of 18 items. Stress coping was assessed using the CRI-A (20). This questionnaire contains 48 items and provides eight scales assessing different coping strategies: logical analysis, positive reappraisal, seeking guidance and support, problem solving, cognitive avoidance, acceptance or resignation, seeking alternative rewards, and emotional discharge. Lastly, personality variables were assessed using the NEO-FFI questionnaire (21); which includes 60 items abbreviated as five major dimensions of personality: neuroticism, extraversion, openness, agreeableness, and conscientiousness.

#### Self-Reported Bruxism Questionnaire

To evaluate bruxism, we used the Pintado et al. questionnaire (25), which consists of six items: (1) Has anyone heard you grinding your teeth at night? (2) Is your jaw ever fatigued or sore on awakening in the morning? (3) Are your teeth or gums ever sore on awakening in the morning? (4) Do you ever experience temporal headaches on awakening in the morning? (5) Are you ever aware of grinding your teeth during the day? (6) Are you ever aware of clenching your teeth during the day?

In addition, three questions were added to assess the sensations of tension or stiffness in the jaw muscles, thereby increasing diagnosis certainty: (1) How would you rate your jaw muscle stiffness or tension at the present time? (2) What was the greatest jaw muscle tension or stiffness felt in the last 6 months? (3) What was the average jaw muscle intensity or stiffness felt during the last 6 months? The questions included a scale similar to the visual analog scale, ranging from 0 to 10 points, where 0 would indicate the “absence of tension” and 10 would mean “the highest possible tension.” Patients were classified as probable awake bruxers when they answered “Yes” to items 5 or 6 in the Pintado questionnaire, which both refer to the awareness of clenching or grinding teeth during wakefulness, and showed a score equal or >4 regarding to the intensity of the tension and stiffness experienced in the last 6 months. Probable sleep bruxers were evaluated based on items 1–4 of Pintado questionnaire, which refer to sleep bruxism.

### Clinical Examinations

Following the questionnaires, the participants underwent clinical examinations. Firstly, we assessed the potential bruxism in each participant by asking about grinding, clenching, presence of headaches, discomfort and/or jaw tension when waking up and during the day. Likewise, for exclusion and control purposes a clinical examination of the temporomandibular joint (TMJ) was also conducted, following DC/TMD axis I criteria (26). Thus, the clinical examination included pain location, incisal relationships, opening pattern, opening movements, lateral and protrusive movements, TMJ noises, joint locking, muscle and TMJ palpation, and supplementary muscles palpation (posterior mandibular area, submandibular region, lateral pterygoid, and temporal tendon).

### Procedure

All participants were dentistry students. After receiving instructions, they filled out the questionnaires at the same time in a quiet environment. Although no time limit was set, it took participants around 60 min on average to complete all the questionnaires. The questionnaires were scheduled so that they were administered outside of university exam periods, which might increase stress levels. Due to space and trained personal limitations that could not be avoided at that moment, the clinical exploration took place 2 months later. They were carried out in the dentistry room of the faculty by 4th year dentistry students under the supervision of three calibrated Odontology faculty teachers of Craniomandibular Dysfunction and Orofacial Pain subjects. Therefore, three calibrated dentists in DC/TMD protocol (co-authors of this study), supervised and validated the adequacy of the students' explorations. It was a double-blind design, since dentists in charge of the clinical examination were not aware of the psychological assessment results and psychologists and participants did not know to which group each participant belonged.

### Statistical Analysis

Since a multivariate study was conducted, sample size was calculated with the method described in Naing, et al. (27), assuming an awake bruxism prevalence of 5% in the general population (2), a level of confidence of 0.95 and a precision of 0.07. Sample size calculation resulted in 19 subjects per group, that is a total of 38 participants. The statistical analyses were calculated using SPSS 24 Statistics Software (IBM) and R, including the package MVN for Mardia's multivariate analysis (28). Items multivariate normality was assessed via Mardia's multivariate kurtosis and skewness coefficients (29). In order to compare the psychological variables between bruxers and control groups, a one-way MANOVA was carried out including direct scores from all the scales of each questionnaire. The response to the three questions included in the self-reported bruxism questionnaire on mandibular tension or stiffness (see detailed description above) was also included in the data analysis.

## Results

The estimates of Mardia's multivariate kurtosis and skewness coefficients were statistically non-significant (949.7, *p* = 0.66 and −1.31, *p* = 0.18, respectively), indicating that normality can be assumed for MANOVA analyses calculations.

**Figure 1:**
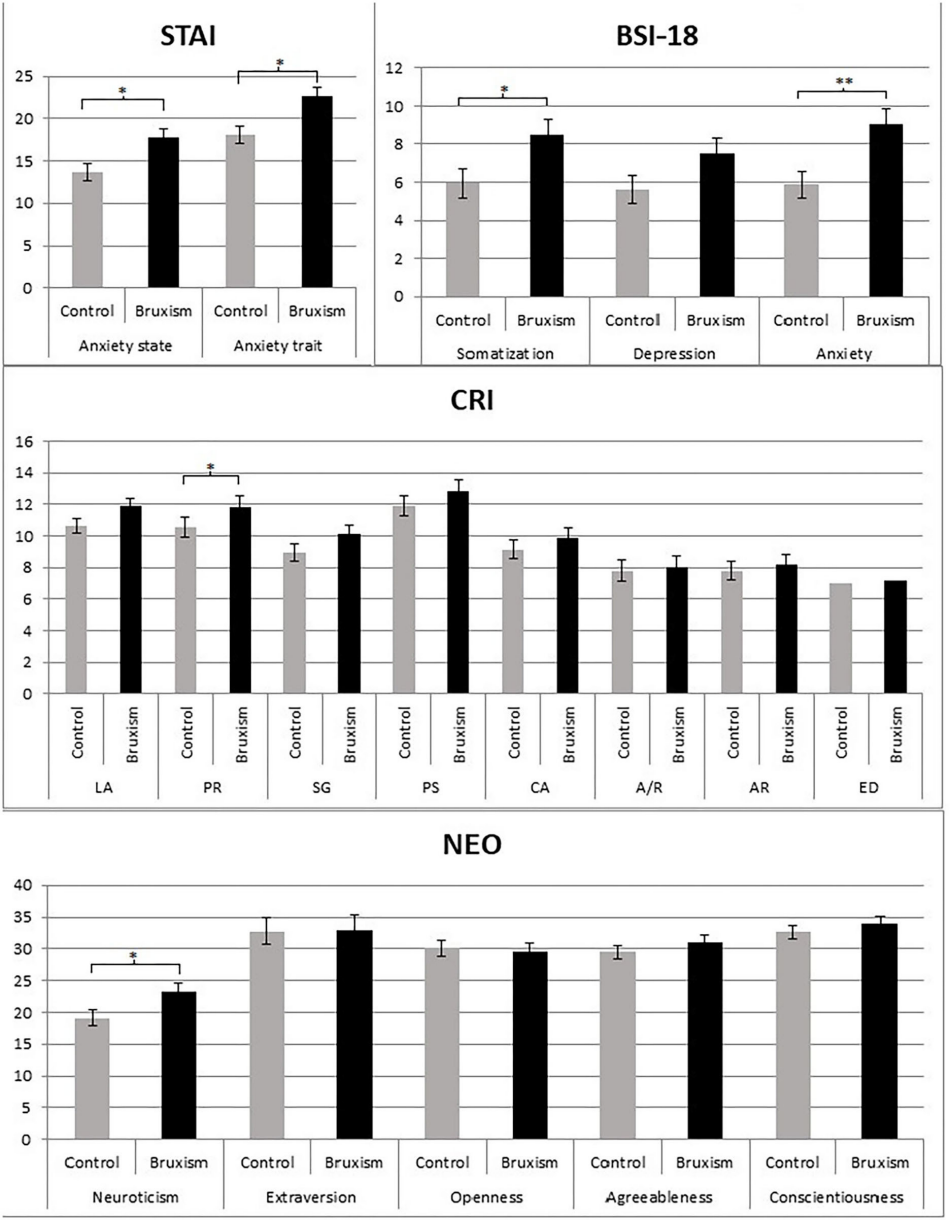
Averages and standard errors of STAI (State and Trait Anxiety Inventory); BSI-18 (Brief Symptom Inventory), CRI-A (Coping Responses Inventory—Adult form), and NEO-FFI (NEO Personality Inventory) scales for control and bruxers groups. Graphics for ST-DEP (State and Trait Depression Inventory) are not included due to the lack of significant results, however, means and statistical analyses can be seen in Table 1. LA, logical análisis; PR, positive reappraisal; SG, seeking guidance and support; PS, problem solving; AV, cognitive avoidance; A/R, acceptance and resignation; AR, seeking alternative rewards; ED, emotional discharge. *p < 0.05; **p < 0.001. Bars represent the standard error of the mean.

State anxiety levels (transient emotional state) were significantly greater (*p* < 0.05) in the awake bruxism group (M = 17.8, *SD* = 6.8) than in the control group (M = 13.7, *SD* = 6.8). Additionally, trait anxiety levels (general propensity to anxiety) were also higher in the awake bruxism group (M = 22.6, *SD* = 7.4) than in the control group (M = 18.1, *SD* = 9.2) (*p* < 0.05). Accordingly, anxiety symptoms, as assessed with the BSI-18 questionnaire, were significantly higher (*p* < 0.01) in the awake bruxism group (M = 9.1, *SD* = 3.9) than in the control group (M = 5.8, *SD* = 4.1). In regard to the somatization scale, significant differences were also present between groups (*p* < 0.05), with higher scores appearing among the awake bruxers participants (M = 8.5, *SD* = 4.8) with in comparison to the healthy ones (M = 5.9, *SD* = 3.9). However, despite the fact that the awake bruxer's group displayed higher depression scores than the control group, (M = 7.5, M = 5.6 and *SD* = 4.4, *SD* = 3.9, respectively) the statistical analysis revealed that this difference was statistically non-significant, showing only a weak trend (*p* = 0.09) (Figure 1). Furthermore, the ST-DEP depression questionnaire failed to show significant differences between the two groups in any of the scales or subscales. For detailed analyses see Table 1.

**Table 1:** STAI (State and Trait Anxiety Inventory), ST-DEP (State and Trait Depression Inventory), BSI-18 (Brief Symptom Inventory), CRI-A (Coping Responses Inventory—Adult form), and NEO—FFI (NEO Personality Inventory) multiple analyses of variances (MANOVAs).

**Questionnaires**	**Scale**	**Group**	**Mean**	**Std.**	**DF**	***F***	***p***	***ηp*^**2**^**	**θ**
STAI	Anxiety state	Control	13.69	6.77	1.57	5.25	0.03^*^	0.08	0.61
(Anxiety)		Bruxism	17.76	6.78					
	Anxiety trait	Control	18.12	9.24	1.57	4.15	0.04^*^	0.07	0.52
		Bruxism	22.65	7.39					
ST/DEP	Euthymia state	Control	10.88	2.85	1.57	0.07	0.79	0.00	0.06
(Depression)		Bruxism	11.08	2.95					
	Dysthymia trait	Control	6.45	1.87	1.57	0.89	0.35	0.02	0.15
		Bruxism	7.00	2.55					
	Euthymia state	Control	11.24	3.64	1.57	1.17	0.28	0.02	0.18
		Bruxism	10.27	3.16					
	Dysthymia trait	Control	6.73	1.72	1.57	0.34	0.56	0.01	0.09
		Bruxism	7.00	1.85					
BSI-18	Somatization	Control	5.94	3.98	1.57	4.83	0.03^*^	0.08	0.58
(symptoms)		Bruxism	8.46	4.84					
	Depression	Control	5.61	3.96	1.57	2.97	0.09	0.05	0.39
		Bruxism	7.50	4.47					
	Anxiety	Control	5.88	3.86	1.57	9.51	0.00^*^	0.14	0.86
		Bruxism	9.08	4.07					
CRI	Logical Analysis	Control	10.62	2.85	1.57	3.17	0.08	0.05	0.42
(Coping)		Bruxism	11.9	2.59					
	Positive Reappraisal	Control	10.55	2.55	1.57	4.06	0.04^*^	0,07	0.51
		Bruxism	11.85	2.34					
	Seeking Guidance	Control	8.94	3.98	1.57	1.53	0.22	0.03	0.23
		Bruxism	10.08	2.78					
	Problem Solving	Control	11.92	3.26	1.57	1.28	0.26	0.02	0.20
		Bruxism	12.81	2.56					
	Cognitive Avoidance	Control	9.12	3.61	1.57	0.59	0.45	0.01	0.12
		Bruxism	9.85	3.58					
	Acceptance/Resignation	Control	7.79	3.14	1.57	0.06	0.81	0.00	0.06
		Bruxism	8.00	3.65					
	Alternative Reward	Control	7.77	3.88	1.57	0.15	0.70	0.00	0.07
		Bruxism	8.15	3.56					
	Emotional Discharge	Control	6.97	3.72	1.57	0.05	0.81	0.00	0.06
		Bruxism	7.17	2.66					
NEO	Neuroticism	Control	19.09	7.31	1.57	5.14	0.03^*^	0.08	0.61
(Personality)		Bruxism	23.27	6.65					
	Extraversion	Control	32.79	14.46	1.57	0.00	0.95	0.00	0.05
		Bruxism	33.00	8.09					
	Openness	Control	30.12	7.49	1.57	0.12	0.73	0.00	0.06
		Bruxism	29.46	6.92					
	Agreeableness	Control	29.48	6.61	1.57	0.87	0.35	0.01	0.15
		Bruxism	31.04	5.98					
	Conscientiousness	Control	32.64	6.148	1.57	0.58	0.45	0.01	0.12
		Bruxism	33.88	6.37					

*Std., Standar deviation; DF, degree of freedom; ηp^2^, Effect size; θ, power*.

* *< 0.05*.

Regarding the coping questionnaire (CRI-A), of eight scales analyses revealed significant differences between the groups in positive reappraisal (*p* < 0.05) and a tendency toward logical analysis (*p* = 0.08), suggesting that awake bruxer participants used more positive reappraisal strategies (RP) and logical analysis (AL) than healthy control participants (RP: M = 8.1, M = 10.5, and *SD* = 2.3, *SD* = 2.5, respectively; AL: M = 11.9, M = 10.6, and *SD* = 2.6 *SD* = 2.8, respectively). The remaining coping scales did not show any significant difference (Table 1, Figure 1).

The analysis of the Personality Questionnaire (NEO) yielded statistically significant results for neuroticism (*p* < 0.05), specifically indicating that the level of neuroticism was higher for the awake bruxism group (M = 23.3, *SD* = 7.3) than for the control group (M = 19.1, *SD* = 6.9). Other personality dimensions failed to yield significant differences between the two groups.

Finally, in the self-reported bruxism questionnaire, there were significant differences on all the items regarding stiffness intensity and muscular tension, affirming that the awake bruxers scored significantly higher in these variables: At the questionnaire moment (*p* < 0.01) (Case group: M = 4.7, *SD* = 2.2; Control group: M = 2.0, *SD* = 1.9), maximum in the last 6 months (*p* < 0.01) (Case group: M = 7.2, *SD* = 1.6; Control group: M = 4.0, *SD* = 2.7), and average over the last 6 months (*p* < 0.01) (Case group: M = 5.0, *SD* = 1.6; Control group: M = 2.6, *SD* = 2.3).

## Discussion

The awake bruxer group showed significantly higher levels of state and trait anxiety, symptoms of anxiety, symptoms of somatization and neuroticism than the control group. With respect to coping strategies, the awake bruxer participants used positive reappraisal to a greater extent (Figure 1).

The data are consistent with previous studies that found an association between awake bruxism and anxiety, sensitivity to stress, and various personality factors (30, 31). As in the present study, most of these investigations adopted a clinical or self-reported diagnosis of bruxism. According to Loobezoo et al. (16), the fact that bruxism is significantly associated with some psychological conditions such as stress and anxiety (both assessed using validated methods) as well as muscle and joint pain, makes self-reported bruxism worthy of further exploration. Nonetheless, in the present study we tried to add self-reports by including three questions about tension or stiffness in the jaw muscles on a 0–10 scale. In addition, during clinical inspection participants were assessed again in order to improve the reliability and validity of the probable bruxism diagnosis as much as possible. Nonetheless electromyographic assessment would have helped to increase diagnosis certainty.

Although anxiety appears to be the most consistent psychological factor involved in awake bruxism, questionnaires that differentiate between state (transient) and trait (general propensity) anxiety are seldom used. The significant relationship between state anxiety and awake bruxism might reflect a very common observation in everyday clinical practice, which is noted when patients are questioned about the presence of mandibular tension or tightening: they often answer that it is not always present, but it is frequent in stressful situations. This observation indirectly supports the notion of bruxism as a continuum without a clear cut-off point between neutral or beneficial and pathological behavior (1). In addition to the suggested beneficial function of bruxist behavior during sleep, for example, in mediating the recovery from respiratory arousals or reducing teeth wear due to gastro-oesophageal reflux by increasing salivation, bruxism during the day seems to be inherently related to facial emotional displays. Darwin in the book “The Expression of the Emotions in Man and Animals” already stated that “the grinding of the teeth, and the uttering of piercing shrieks, all give relief under an agony of pain” (32). More recently, animal models pointed out that chewing might reduce stress response, since biting on a wooden stick during a restraint situation reduced blood pressure, core temperature, suppression of hippocampal long-term potentiation, and serum chemical mediators of stress (33–36). Additionally, evidence in humans also supports the notion that mastication might reduce negative mood, cortisol release, and the production of salivary chromogranin, a marker of mental stress that reflects sympathetic activity (37–39). As a result, it has been hypothesized that bruxism might play a role in stress reduction (40, 41). It is well-known that emotional expression, including face expression, are preferable to their repression in relation to stress, particularly acute and chronic pain severity is related to anger inhibition (42). In fact, many stress reduction programs use techniques favoring emotional expression (43).

In our study we find significantly elevated levels of trait anxiety in our awake bruxer sample, as opposed to other authors (44), who failed to find such significant differences although their patients did show higher levels than the controls in this variable. This difference may be explained by the use of different cut-off points for the selection of participants. Thus, it is possible that our subjects, who were selected based on both the clinical exploration and the self-reported questionnaire, comprised a more constricted sample, avoiding the inclusion of doubtful or milder cases. Whatever the case, it has been shown that people with elevated trait anxiety tend to perceive situations as more threatening or stressful, therefore favoring the more frequent presence of awake bruxism. The higher levels of neuroticism found in the personality questionnaire and in previous research (45), could be interpreted in a similar manner as the neuroticism personality trait is characterized by emotional instability, including tendency for anxiety and excessive preoccupation over daily situations, which could, in turn, relate to the presence of awake bruxism. Thus, more research is needed which takes into account the different degrees of bruxism and their relation to psychosocial factors.

Previous studies have found high levels of depression and somatization in awake bruxer patients while our data showed only a weak tendency for higher depression in these patients. The depression variable is not always included in studies on bruxism and, when specific questionnaires are used, as in our case, higher levels of depression do not always appear (46). As in the case of anxiety, the strict inclusion criteria, a different study population and other sample characteristic such as age may explain the divergent data.

Finally, the awake bruxer participants showed higher levels of adaptive coping strategies like positive reappraisal. Positive reappraisal is a strategy used to cope with negative events by attempting to see a problem in a positive way while still accepting the reality of the situation (20). This strategy is generally considered as an adaptive cognitive strategy in stress coping models. However, TMD patients tend to show negative stress coping strategies (47, 48). While awake bruxers displayed higher levels of positive coping strategies, TMD patients would use more negative ones. On the one hand, it is possible that awake bruxism may be playing a positive psychological role in those patients, allowing them to partly discharge some of the psychological tension (40, 41), which would enable them to display more adaptive coping strategies. On the other hand, recent investigations demonstrate that arousal reappraisal (encouraging individuals to interpret heightened physiological arousal as a tool that can help maximize performance) benefits cardiovascular and cognitive responses to stress (49, 50). Furthermore, short-term stress responses can enhance immune function (51). Therefore, larger levels of positive reappraisal in awake bruxism might indeed prevent the adverse effects of stress on health. It is possible that awake bruxism could constitute a risk factor for TMD only when accompanied by maladaptive coping strategies and/or the lack of adaptive ones (positive reappraisal) that might increase perceived stress, which, in turn, has been described as the strongest psychological predictor of TMD (52). Logical Analysis showed a trend toward significance, so it is possible that in larger samples, other adaptive strategies besides positive reappraisal could emerge. Therefore, more research in awake bruxist and TMD patients is needed in order to clarify the validity of this hypothesis and to allow for an adequate understanding and treatment of both conditions.

The awake bruxism group showed significantly higher scores in anxiety (state and trait), somatization and neuroticism than the control group. Our data are in line with the majority of previous studies. In relation to stress coping, the awake bruxism group showed higher levels of positive reappraisal (adaptive coping strategy), which might prevent negative effects of stress on health, such as the onset or worsening of TMD. Furthermore, when adaptive coping strategies are present, awake bruxism may be playing a positive psychological role, allowing for a partial discharge of some of the psychological tension, as some authors hypothesize (40, 41). In this line, chewing gum alleviates negative mood and reduces cortisol during acute laboratory psychological stress (37). Furthermore, according to Ono's review (2010) mastication during stress conditions in animal models, might increase stress-induced hippocampal neurogenesis, synaptic plasticity, and cognitive function by attenuating stress hormones and their receptors by activating serotonin neurons in the dorsal raphe nucleus (41).

Although, data support the implication of psychological factors in bruxism, more research is needed acknowledging the dimensional nature of bruxism, distinguishing between definitive sleep and awake subtypes, and relating them to the various psycho-sociological factors, including coping strategies. In addition, the sample selection (a cohort of university students) favored the homogeneity of the samples in terms of age, sociological, cultural and environmental variables. However, further research including a larger and more representative sample of participants (not only students), selected also using electromyographic assessment could enhance the generalizability of the results and increase diagnosis certainty. Additionally, clinical and psychological explorations should be done at the same time. Finally, it should be noticed that the proportion of males and females per group did not match exactly. Although, a proportion analyses did not find significant differences in the ratio of males to females between cases and controls, further research may benefit from using paired samples with an equal number of males and females, since gender is an important variable that should be controlled in psychological assessment.

In conclusion, despite the limitations of the study, the findings may have clinical significance. It was observed that bruxers showed larger levels of anxiety, somatization, and neuroticism, similarly to previous studies on TMD patients. Nonetheless, they also displayed more adapted coping strategies while, frequently, TMD patients tend to present less adaptive coping styles. Thus, awake bruxism might play a positive role in stress coping, which would be compatible with the hypothesis of mastication as a means of relieving psychological tension. This hypothesis should be further validated by future research comparing TMD patients with definitive awake bruxers and controls using larger and more representative samples.

## Data Availability Statement

The raw data supporting the conclusions of this article will be made available by the authors, without undue reservation.

## Ethics Statement

The studies involving human participants were reviewed and approved by Comité Ético de Investigación Clínica Hospital Clínico San Carlos. The patients/participants provided their written informed consent to participate in this study.

## Author Contributions

LJ-O, IA-G, and TS-S designed, directed the experiment, participated in patient assessment, and data discussion. XS-G, LB-G, FA, and DM-C participated in the clinical explorations, data collection, and data processing. LJ-O, FA, and XS-G also collaborated in the writing and review process of the article. All authors contributed to the article and approved the submitted version.

## Conflict of Interest

The authors declare that the research was conducted in the absence of any commercial or financial relationships that could be construed as a potential conflict of interest.
